# Texture analysis of *T*_1_- and *T*_2_-weighted MR images and use of probabilistic neural network to discriminate posterior fossa tumours in children

**DOI:** 10.1002/nbm.3099

**Published:** 2014-04-13

**Authors:** Eleni Orphanidou-Vlachou, Nikolaos Vlachos, Nigel P Davies, Theodoros N Arvanitis, Richard G Grundy, Andrew C Peet

**Affiliations:** a Birmingham Children's Hospital NHS Foundation TrustBirmingham, UK; b School of Cancer Sciences, College of Medical and Dental Sciences, University of BirminghamEdgbaston, Birmingham, UK; c Department of Medical Physics, University Hospitals Birmingham NHS Foundation TrustEdgbaston, Birmingham, UK; d Institute of Digital Healthcare, WMG, University of WarwickCoventry, UK; e Children's Brain Tumour Research Centre, Queens Medical Centre, University of NottinghamUK

**Keywords:** texture analysis, MRI, paediatric posterior fossa tumours

## Abstract

Brain tumours are the most common solid tumours in children, representing 20% of all cancers. The most frequent posterior fossa tumours are medulloblastomas, pilocytic astrocytomas and ependymomas. Texture analysis (TA) of MR images can be used to support the diagnosis of these tumours by providing additional quantitative information. MaZda software was used to perform TA on *T*_1_- and *T*_2_-weighted images of children with pilocytic astrocytomas, medulloblastomas and ependymomas of the posterior fossa, who had MRI at Birmingham Children's Hospital prior to treatment. The region of interest was selected on three slices per patient in Image J, using thresholding and manual outlining. TA produced 279 features, which were reduced using principal component analysis (PCA). The principal components (PCs) explaining 95% of the variance were used in a linear discriminant analysis (LDA) and a probabilistic neural network (PNN) to classify the cases, using DTREG statistics software. PCA of texture features from both *T*_1_- and *T*_2_-weighted images yielded 13 PCs to explain >95% of the variance. The PNN classifier for *T*_1_-weighted images achieved 100% accuracy on training the data and 90% on leave-one-out cross-validation (LOOCV); for *T*_2_-weighted images, the accuracy was 100% on training the data and 93.3% on LOOCV. A PNN classifier with *T*_1_ and *T*_2_ PCs achieved 100% accuracy on training the data and 85.8% on LOOCV. LDA classification accuracies were noticeably poorer. The features found to hold the highest discriminating potential were all co-occurrence matrix derived, where adjacent pixels had highly correlated intensities. This study shows that TA can be performed on standard *T*_1_- and *T*_2_-weighted images of childhood posterior fossa tumours using readily available software to provide high diagnostic accuracy. Discriminatory features do not correspond to those used in the clinical interpretation of the images and therefore provide novel tumour information. Copyright © 2014 John Wiley & Sons, Ltd.

## INTRODUCTION

Brain and spinal cord tumours are the most common solid tumours in children (1,2), representing 20% of all childhood cancers (3). Brain tumours also cause the highest number of deaths among paediatric cancer patients (4), and the combination of the tumour and its treatment commonly causes significant morbidity in terms of physical deficits, neuropsychological and neuroendocrine effects (5).

Conventional MRI is collected on all cases of paediatric brain tumour, and is usually assessed qualitatively; quantitative analysis is not undertaken routinely. However, there is a potential wealth of information not visible to the human eye, which can be extracted from MR images and quantified using texture analysis (TA) techniques. This may yield additional information to characterise paediatric brain tumours, provide new insights on the characteristic features and enable the combination of MRI and data from other quantitative techniques, such as MRS.

In this study, TA was employed for the diagnosis of paediatric posterior fossa tumours. Posterior fossa tumours are studied here, as they are relatively common and usually biopsied, therefore providing a useful test-bed for the technique. The most common posterior fossa tumours are pilocytic astrocytomas (grade 1), medulloblastomas (grade 4) and ependymomas (grade 2 or 3) (4). Although posterior fossa tumours are generally resected (except those located in the brainstem) (6), having a means of non-invasive diagnosis is useful in guiding and planning treatment. Conventionally, radiologists use a set of characteristics known to be associated with a particular tumour type and deduce a diagnosis from these (7–9). However, each type of tumour can have variable appearances within the group (10,11), and it is to be expected that the diagnosis from conventional radiological reporting will not be completely accurate. Given the skilled nature of the process, the accuracy of diagnosis will also depend on the training, expertise and judgment of the radiologist. The accuracy of diagnosis by conventional radiological reporting of MRI for paediatric brain tumours has been quoted as 63%, with a further 10% having the correct diagnosis in the differential; expert retrospective review of the images increased the accuracy of diagnosis to 71% (12). Similar rates have been found for posterior fossa tumours in children (8). Diffusion-weighted imaging can provide some extra discrimination between the tumour types, but quantitative analysis shows that there is overlap in the mean apparent diffusion coefficients between the different tumour types (13).

There is increasing interest in whether a more sophisticated quantitative analysis of MRI may add to the conventional reporting. The analysis of diffusion-weighted imaging by a combination of quantitative image analysis and automated pattern recognition has been reported to show high accuracies in the diagnosis of childhood brain tumours (14), and a similar approach has shown a high diagnostic accuracy for MRS (15). Less attention has been paid to the application of these quantitative pattern recognition techniques to the *T*_1_- and *T*_2_-weighted images collected as part of routine clinical practice. TA provides a method for the quantification of the variation in image patterns, which can also include data not taken into account in conventional radiological reporting because of its small scale (7). The quantitative and largely automated nature of the technique may be particularly valuable, and its combination with automated pattern recognition may provide a powerful method for the quantitative analysis of conventional images. The availability of conventional MRI and analysis packages means that no additional infrastructure, expertise or cost is required, and the technique can potentially be widely implemented.

TA has shown promise in the discrimination between lesions on MR images (7) and provides quantitative, reproducible results. It has been used in brain tumours, epilepsy, multiple sclerosis and other disorders (7,16,17), as well as for various image types (e.g. MR, X-ray) (18–25). TA involves four issues: feature extraction, texture discrimination, texture classification and shape from texture (26).

In this study, feature extraction and texture classification are used. There are many textural features that can be investigated and various ways of computationally implementing the analysis. One of the methods, which has been used in research and is well documented in the literature, is MaZda software (25,27,28), a software package for two- and three-dimensional image TA; this was used in this study. There are four approaches to TA, namely structural, statistical, model based and transform methods (26).

Another issue in TA is to ensure that the features computed characterise the image texture exclusively, regardless of the global image characteristics, such as overall brightness, contrast or other bias (25). This is achieved in MaZda using a normalisation procedure, which reduces the dependence of higher order parameters on first-order gray-level distribution. Previous studies have shown that TA can provide reproducible results under different MRI acquisition protocols if appropriate normalisation is used. (29–31).

## CLASSIFICATION

The general purpose of feature selection is to find the optimum combination of features which provides the best classification result. Features that are not relevant to the classification problem should be eliminated. Principal component analysis (PCA) is an unsupervised feature reduction technique, which is widely used in pattern recognition studies (32–36).

Various types of artificial neural network (ANN) have found widespread use in a great number of medical diagnostic decision support system applications, as they are believed to be efficient and reliable algorithms with a great predictive power, when compared with other statistical modelling techniques, such as statistical regression (37–41). Probabilistic neural networks (PNNs) can provide a very general and powerful classification model when there are adequate data of known classification. PNNs offer several advantages over back-propagation networks, such as shorter training time (42), effectiveness on small datasets, and easier and better interpretability (37,43,44). Moreover, PNNs allow true incremental learning, where new training data can be added at any time, without requiring re-training of the entire network (44–49). The explanation of PNN parameters that can influence classification has been described previously (37,44,50,51). In this study, we combine TA with PNN to provide a quantitative method for decision support in the non-invasive diagnosis of childhood posterior fossa tumours.

## METHODS

### MRI

Children with pilocytic astrocytomas, medulloblastomas and ependymomas of the posterior fossa, who had MRI at Birmingham Children's Hospital prior to treatment (except stereotactic biopsy), were included in the study.

#### Acquisition protocol

Images were acquired on a Siemens (Germany) Symphony 1.5-T ΜR scanner. *T*_1_-weighted (pre-contrast) axial two-dimensional turbo spin echo images were acquired using a TR of 700 ms and a TE of 10–20 ms. *T*_2_-weighted axial two-dimensional turbo spin echo images were acquired using a TR of 3000 ms and a TE of 100 ms. The images were anonymised and stored, in DICOM format, on a research database, available at the Institute of Child Health's Brain Tumour Research Group. Radiology reports made at the time of MRI were reviewed for all cases to identify whether a diagnosis had been proposed. Radiologists would have had access to clinical information and all radiological information available at the time when making the report.

### TA

**Figure 1:**
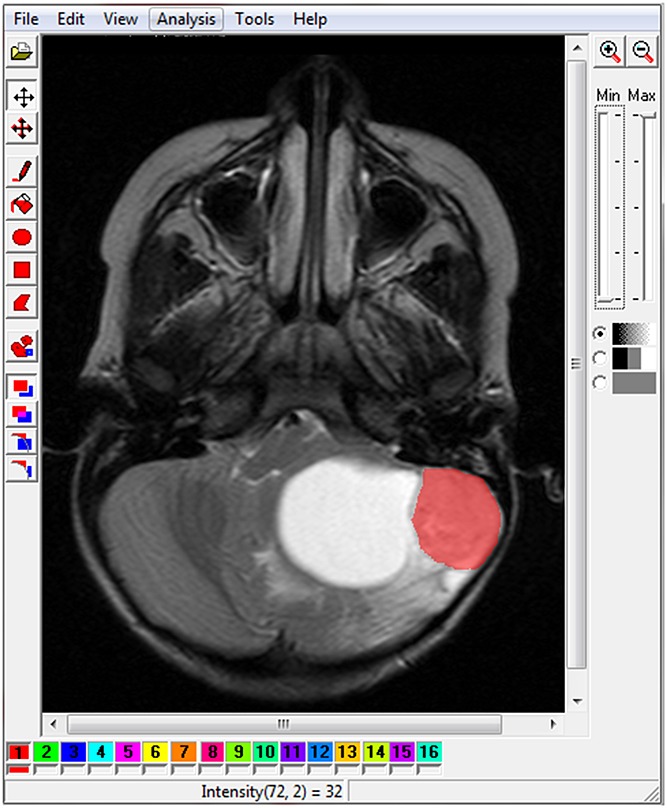
Region of interest (ROI) in red loaded over image in MaZda after image normalisation. The tumour is a pilocytic astrocytoma with a characteristic peripheral solid nodule and large cyst. It should be noted that the large cyst is not included in the ROI.

*T*_2_ images were analysed first, as tumours were generally easier to detect on these, and three image slices per patient were chosen, where the tumour was most visible/largest. Three images were used per patient in order to account for intra-tumour variability and increase the amount of data available on the MR image that was used for TA. The tumour or region of interest (ROI) was chosen in Image J using thresholding ± manual outlining. The image slices and ROI were selected as a consensus from the multi-disciplinary team. The ROI was saved in .bmp format, opened in MaZda (v. 3.3) (25,27,28) and saved in .roi format. For *T*_1_ images, the slices most closely matching the *T*_2_ slices were used, and the same ROI was employed. In MaZda, the image was viewed, normalised, the ROI was loaded over it and TA was run (Fig. 1). Normalisation was performed by choosing the intensity range of each ROI to be within three standard deviations below and above the mean.

TA yielded 279 features based on histogram, gradient map, co-occurrence matrix produced for four directions and five inter-pixel distances, run length matrix, autoregressive model and Harr wavelet transform. Briefly, histogram-based features refer to statistical parameters of the intensity of pixels within the ROI. Gradient map-based features use the histogram of the image gradient and a feature set is calculated for the image intensity distribution. The co-occurrence matrix is a second-order histogram, which is calculated from the intensities of pairs of pixels, with the spatial relationship of the pairs of pixels defined. Run length matrix features refer to counts of pixel runs with the specified grey-scale level and length (performed for four directions). The autoregressive model assumes that the pixel intensity may be predicted as a weighted sum of four neighbouring pixel intensities (with reference to the mean value of the image ntensity). The model's parameters are the weights associated with these pixels and the variance of the minimised prediction error. Finally, in the Harr wavelet transform method, the wavelet images are scaled up to five times, transforming the image into 20 frequency channels. The texture-characterising features arise from data on texture frequency components extracted from the energies computed within the channels.

### Feature reduction

PCA was applied in Minitab 15 (52), a commercially available statistics package, in order to identify the underlying data structure and to achieve dimensionality reduction. The number of principal components (PCs) to be used in the classifiers was chosen in order to account for 95% of the cumulative variance.

### Classification

Classification of the posterior fossa tumours based on the MR image features was performed using the PC scores as input variables in a PNN and linear discriminant analysis (LDA) in DTREG v.9.6 (53), a commercially available software. Different PNN training schemes can be examined using the commercial software DTREG: (i) a Gaussian kernel or a reciprocal kernel; and (b) one smoothing parameter *σ* for all input random variables or a different *σ* for each input random variable or a separate *σ* for each input random variable and class. A Gaussian kernel and a separate *σ* for each input random variable and class were used as the training scheme, as these gave the best results. Smoothing parameters may take values of different orders of magnitude. As these parameters have a great effect on the classification performances of PNN, their optimal values were automatically assessed. The PNN had 51 and 43 hidden neurons in the second layer for *T*_1_ and *T*_2_, respectively, and 84 hidden neurons for the combined *T*_1_ and *T*_2_ classifier, determined using the minimum error criterion; three summation layer neurons; and one output layer neuron. Classification errors were determined using the known class labels from histopathology. As a result of the small numbers of data (40 patients), the classifier was validated using leave-one-out cross-validation (LOOCV) [leaving one patient out at a time (three images)]. Ten-fold cross-validation was also performed to further test the reliability of the classifier in terms of over-fitting errors.

## RESULTS

MR images from 21 patients with medulloblastomas, 14 patients with pilocytic astrocytomas and five patients with ependymomas were investigated using TA. In the conventional radiological review, 11 of 21 (52%) medulloblastomas, one of five (20%) ependymomas and six of 14 (43%) pilocytic astrocytomas had the correct diagnosis specified as the most likely, giving an overall accuracy of 18 of 40 (45%). In four of these cases, alternative possible diagnoses were proposed, showing some degree of diagnostic uncertainty. One medulloblastoma was incorrectly diagnosed as an ependymoma and one ependymoma was incorrectly diagnosed as a medulloblastoma. Two tumours in each category had two or more diagnoses proposed with no preference given. In six medulloblastomas, one ependymoma and six pilocytic astrocytomas, no diagnosis or differential was proposed. Scrutiny of the reports for these cases showed that tumour features were often present which could cause difficulty in diagnosis, and most reports were from radiologists who had proposed a diagnosis for other tumours, implying that diagnostic uncertainty existed, rather than a bias towards certain radiologists systematically not reporting a diagnosis. However, if these cases are excluded from the analysis, the accuracy of diagnosis is 11 of 15 (73%) for medulloblastomas, one of four (25%) for ependymomas and six of eight (75%) for pilocytic astrocytomas, giving 18 of 27 (67%) overall. The sensitivities and specificities for each tumour category are given in Table 1.

**Table 1:** Sensitivity and specificity of radiological diagnosis for each tumour category

	Sensitivity (%)	Specificity (%)
Medulloblastoma	58	47
Ependymoma	20	55
Pilocytic astrocytoma	43	75

**Figure 2:**
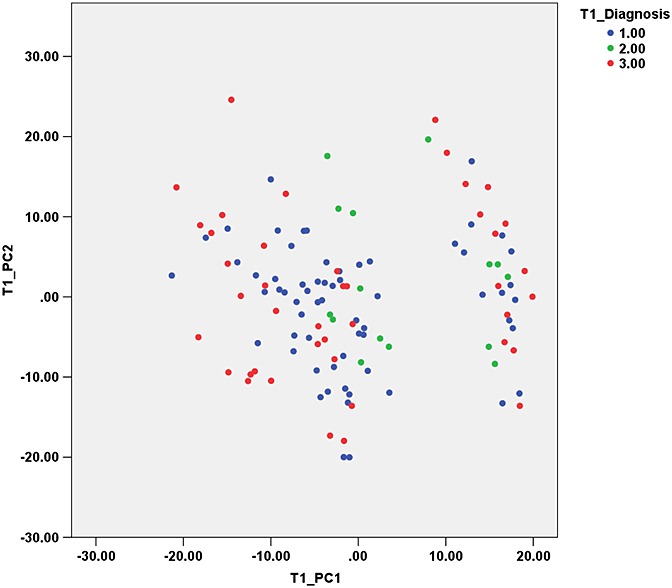
Principal component 2 (PC2) *versus* PC1 from texture features of *T*_1_-weighted images. Groups: 1, medulloblastoma; 2, ependymoma; 3, pilocytic astrocytoma.

PCA of texture features from *T*_1_-weighted images yielded 13 PCs to explain >95% of the variance. The first PC (PC1) mostly represents co-occurrence matrix-derived parameters (Sum Entropy): S(1,0)SumEntrp, S(0,1)SumEntrp. PC2 mostly represents co-occurrence matrix-derived parameters (Correlation): S(4,0)Correlat, S(3,–3)Correlat, S(2,–2)Correlat [equations for the calculation of these parameters can be found in ref. (54)]. *S*(*x*,*y*) represents the co-occurrence matrix for inter-pixel distance *x* along the rows and *y* along the columns. A plot of PC2 *versus* PC1 is shown in Fig. 2, demonstrating the difficulty in separating the three tumour groups based on the PCs alone.

These PCs were then used as input for the PNN to classify the tumours into the three categories (pilocytic astrocytoma, ependymoma and medulloblastoma). The classifier achieved 100% accuracy on training the data and 90% on LOOCV (validation data accuracy: medulloblastoma, 96.8%; ependymoma, 60%; pilocytic astrocytoma, 90.5%). Ten-fold cross-validation gave the same training and validation accuracies with the number of hidden neurons increasing to 120. The sensitivity and specificity were calculated for each category, treating the category of interest as the positive category and grouping the other two categories as negative. The results are shown in Table 2.

**Table 2:** Sensitivity and specificity of probabilistic neural network (PNN) classification at leave-one-out cross-validation for each tumour category

	*T*_1_		*T*_2_		*T*_1_ + *T*_2_	
	Sensitivity (%)	Specificity (%)	Sensitivity (%)	Specificity (%)	Sensitivity (%)	Specificity (%)
Medulloblastoma	96.8	84.2	95.2	91.2	94.4	81.6
Ependymoma	60.0	100	80.0	98.1	63.3	98.1
Pilocytic astrocytoma	90.5	96.2	95.2	98.7	81.0	94.2

**Figure 3:**
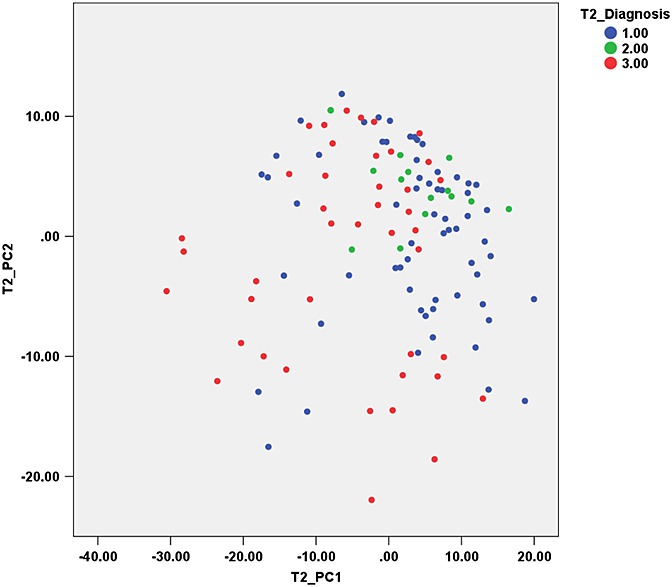
Principal component 2 (PC2) *versus* PC1 from texture features of *T*_2_-weighted images. Groups: 1, medulloblastoma; 2, ependymoma; 3, pilocytic astrocytoma.

Using the same PCs in an LDA resulted in noticeably poorer classification accuracy. The classifier achieved 62.5% accuracy on training the data and 37.5% on LOOCV (validation data accuracy: medulloblastoma, 36.5%; ependymoma, 6.7%; pilocytic astrocytoma, 50.0%). The sensitivity and specificity are reported in Table 3 and the canonical discriminant function scores are presented in Fig. 3.

**Table 3:** Sensitivity and specificity of linear discriminant analysis (LDA) classification at leave-one-out cross-validation for each tumour category

	*T*_1_		*T*_2_		*T*_1_ + *T*_2_	
	Sensitivity (%)	Specificity (%)	Sensitivity (%)	Specificity (%)	Sensitivity (%)	Specificity (%)
Medulloblastoma	36.5	63.2	58.7	82.5	44.4	61.4
Ependymoma	6.7	70.5	13.3	70.5	26.7	71.9
Pilocytic astrocytoma	50.0	70.5	71.4	87.2	45.2	77.6

**Figure 4:**
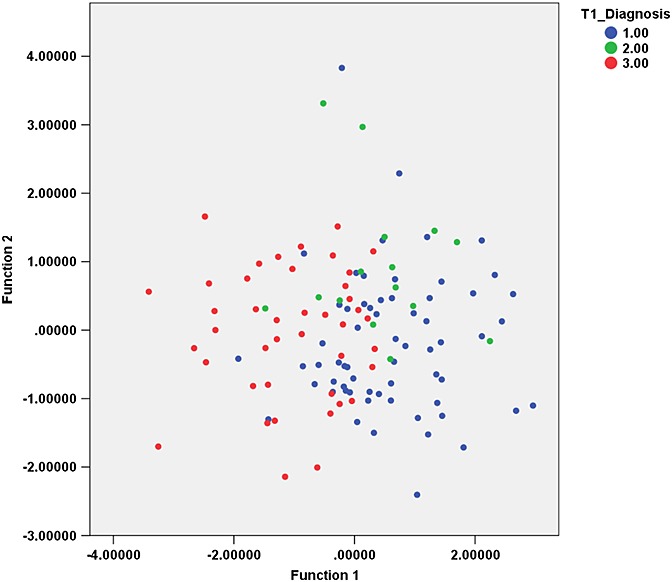
Canonical discriminant functions for texture analysis of *T*_1_-weighted images. Groups: 1, medulloblastoma; 2, ependymoma; 3, pilocytic astrocytoma.

PCA of texture features from *T*_2_-weighted images also yielded 13 PCs to explain >95% of the variance. The first PC (PC1) mostly represents co-occurrence matrix-derived parameters (Difference entropy): S(1,–1)DifEntrp, S(2,–2)DifEntrp, S(0,2)DifEntrp. PC2 mostly represents co-occurrence matrix-derived parameters (Sum variance): S(0,2)SumVarnc, S(0,1)SumVarnc, S(1,–1)SumVarnc [equations for the calculation of these parameters can be found in ref. (54)]. *S*(*x*,*y*) represents the co-occurrence matrix for inter-pixel distance *x* along the rows and *y* along the columns. A plot of PC2 *versus* PC1 is shown in Fig. 4, demonstrating the difficulty in separating the three tumour groups based on the PCs alone.

These PCs were then used as input for the PNN to classify the tumours into the three categories. The classifier achieved 100% accuracy on training the data and 93.3% on LOOCV (validation data accuracy: medulloblastoma, 95.2%; ependymoma, 80%; pilocytic astrocytoma, 95.2%). Ten-fold cross-validation gave a training accuracy of 100% and an overall accuracy for LOOCV of 88.3% (validation data accuracy: medulloblastoma, 93.7%; ependymoma, 66.7%; pilocytic astrocytoma, 88.1%), with the number of hidden neurons increasing to 120. The sensitivity and specificity are reported in Table 2.

**Figure 5:**
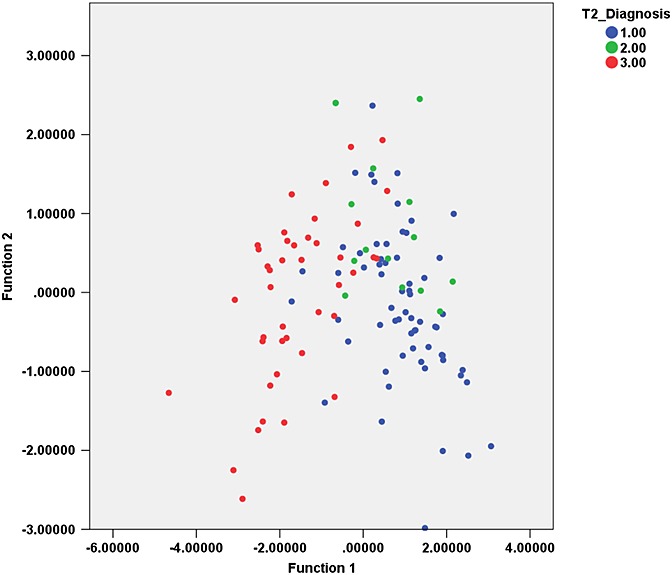
Canonical discriminant functions for texture analysis of *T*_2_-weighted images. Groups: 1, medulloblastoma; 2, ependymoma; 3, pilocytic astrocytoma.

Using the same PCs in an LDA resulted in noticeably poorer classification accuracy. The classifier achieved 68.3% accuracy on training the data and 57.5% on LOOCV (validation data accuracy: medulloblastoma, 58.7%; ependymoma, 13.3%; pilocytic astrocytoma, 71.4%). The sensitivity and specificity are reported in Table 3 and the canonical discriminant function scores are presented in Fig. 5.

PNN and LDA classification were also performed using the combined data from *T*_1_ and *T*_2_ to establish whether the additional information improves the results further. The PNN classifier achieved 100% accuracy on training the data and 85.8% on LOOCV (validation data accuracy: medulloblastoma, 94.4%; ependymoma, 63.3%; pilocytic astrocytoma, 81.0%). Ten-fold cross-validation gave a training accuracy of 100% and an overall accuracy of 85.4% (validation data accuracy: medulloblastoma, 92.9%; ependymoma, 60%; pilocytic astrocytoma, 83.3%), with the number of hidden neurons increasing to 240. The sensitivity and specificity for the PNN classifier are reported in Table 2. The LDA classifier gave much lower classification accuracies, namely 56.7% on training the data, and 42.5% on LOOCV (validation data accuracy: medulloblastoma, 44.4%; ependymoma, 26.7%; pilocytic astrocytoma, 45.2%). The sensitivity and specificity for the LDA classifier are reported in Table 3.

## DISCUSSION

This study shows that TA can be implemented easily on standard *T*_1_- and *T*_2_-weighted images routinely acquired when children present with suspected brain tumours of the posterior fossa. The analysis can be performed with commercially available software, with extensive manuals to support its use, and therefore does not require highly specialised computing knowledge. In addition, it is possible to distinguish between the three most common paediatric posterior fossa tumours with high accuracy (90.0% and 93.3% for *T*_1_- and *T*_2_-weighted images, respectively), making it potentially a valuable tool to aid diagnosis by contributing information not visible to the radiologist on inspection. Combining data from *T*_1_- and *T*_2_-weighted images does not seem to offer additional benefits to the classification.

A review of conventional radiological reporting showed a lower diagnostic accuracy than that estimated from TA and PNN. However, although the correct diagnosis was specified in only 45% of conventional radiological reports, very few cases (2/40, 5%) had an incorrect diagnosis reported. This implies that there is a large diagnostic uncertainty in conventional radiological reporting of these tumours. Even in cases in which the correct diagnosis was specified, four of 18 (22%) had an alternative proposed. A method which can help to improve the confidence with which the diagnosis can be given from MRI would be a useful aid. It is interesting to note that LDA gave similar diagnostic accuracies to conventional radiological reporting, further demonstrating the diagnostic challenge arising from overlapping imaging features between tumour types, and illustrating the potential value of non-linear classification methods.

Although radiologists are often able to distinguish the type of tumour from inspection of the images, this usually includes a degree of uncertainty, as shown above, and all three tumour types may appear in the differential diagnosis in the radiologist's report. This is because some features can overlap between tumour types, and each type of tumour can also have variable appearances within the group. For example, ependymomas appear isointense to grey matter on *T*_2_-weighted images, like medulloblastomas. Cerebellar astrocytomas can show low signal intensity on *T*_1_-weighted images, as can ependymomas and medulloblastomas (10,11). Arle *et al*. (8) studied 33 children with primitive neuroectodermal tumours, astrocytomas and ependymomas/other tumours of the posterior fossa with long-TE single-voxel spectroscopy. Creatine/*N*-acetylaspartate (Cr/NAA), *N*-acetylaspartate/choline (NAA/Cho) and creatine/choline (Cr/Cho), 10 MRI tumour characteristics, tumour size, and patient age and sex were used in a neural network and compared with predictions made by a neuroradiologist blind to the MRS and histopathology results. The neuroradiologist predicted the tumour type with 73% accuracy, and neural networks with different data combinations as inputs achieved accuracies of 58–95%. The neural network which included all data achieved a prediction accuracy of 94.6%.

The current method relies on manual outlining of the ROI, and this can be both time consuming and open to the interpretation of the radiologist. Automated methods for tumour segmentation are becoming available, but, at present, are not completely robust, especially for complex tumours. Semi-automated methods can reduce the time taken to define the ROI, but are still open to the interpretation of the radiologist.

The texture features found to hold the highest discrimination potential are all co-occurrence matrix derived. The co-occurrence matrix is a second-order histogram, computed from the intensities of pairs of pixels. The spatial relationship of the pixels in a pair is defined. When divided by the total number of neighbouring pixels in the ROI, this matrix becomes the estimate of the joint probability of two pixels, a distance *d* apart along a given direction *θ*, having particular co-occurring values *i* and *j*. The result is a square matrix with dimensions equal to the number of intensity levels in the image, for each distance *d* and orientation *θ* (25,54).

It is worth noting the classification method used here, namely PNN, as most previous studies have used some form of LDA (7). Although LDA is a reasonable approach, and a more straightforward and perhaps more intuitive method, it is important to incorporate and test more advanced methods, which may substantially improve classification, as in this study. Discriminant analysis is a traditional statistical classification method built on the Bayesian decision theory, where the posterior probability for the classification decision must be calculated by assuming an underlying probability model. A disadvantage of applying the simple Bayes decision rule is that the density functions are usually not known or cannot be assumed to be normal, and therefore the posterior probabilities cannot be determined directly. In contrast, neural networks estimate the posterior probabilities directly, the basis for establishing a classification rule. Although statistical pattern classifiers are based on Bayes decision theory and posterior probabilities, thus linking them to neural networks, a direct comparison between them may not be possible, as neural networks are non-linear model-free methods, whereas statistical methods are linear and model based (55). Neural networks have an advantage over conventional classification methods for a number of other reasons. They are data-driven self-adaptive methods, which can adjust to the data without explicit specification of functional or distributional form for the underlying model. They can also approximate any function arbitrarily closely. Furthermore, they are non-linear models, and are thus flexible in modelling complex relationships (55). LDA has been shown to perform well when datasets are linearly separable, but classification accuracies decrease when non-linearity is present. However, with real datasets, the degree of non-linearity is often not known; thus the use of both LDA and neural networks to compare performance is useful (56). Linear classifiers may not have the power to learn the underlying relationships sufficiently well, resulting in under-fitting of the data (55).

Classification performance tends to increase with increasing number of hidden nodes, but results in increasing computation cost. This may be important in complex applications, where best validity and computational cost factor need to be balanced (57). In this study, we had a relatively small sample size and therefore focused on improving the classification results and their generalisability in order to demonstrate the value of TA in the diagnosis of paediatric posterior fossa tumours. The technique is used on this test-bed of tumours and can be extended to larger datasets and other types of brain tumour classifiers. The size of neural networks can be decreased when there are larger datasets, and even more so when these datasets are large compared with the complexity of the decision surface (58).

Posterior fossa tumours are a good test-bed for the technique as they are easy to identify on imaging, and usually belong to one of the three categories mentioned. TA and classification could also be applied to other brain tumours, such as low-grade gliomas, using the analysis scheme described in this study, and the results combined with MRS in the same cohort. In addition, TA can be used in prognostication and treatment monitoring to provide additional, robust and reproducible information. An important advantage of quantitative analysis, such as that presented here, is the ability to combine results with other quantitative techniques, such as MRS and diffusion imaging.

## CONCLUSION

This study shows that TA can be implemented easily on standard *T*_1_- and *T*_2_-weighted images, routinely acquired when children present with suspected brain tumours of the posterior fossa. Discriminatory features are mainly associated with small-scale structure and thus do not correspond to the macroscopic features used in the clinical interpretation of the images. Therefore, TA can provide novel tumour characteristics. This methodology can be extended to other paediatric brain tumours, such as low-grade gliomas, and combined with MRS metabolite values in a pattern recognition method. Information from the two techniques is likely to be independent and the combination could provide improved characterisation. TA can be performed with commercially available software, with extensive manuals to support its use, and therefore does not require highly specialised computing knowledge. TA can produce a quantitative analysis of MR images which can aid in the diagnosis of childhood posterior fossa tumours and could play an important role in the radiological investigation of these patients.

## Abbreviations

ANN: artificial neural network
LDA: linear discriminant analysis
LOOCV: leave-one-out cross-validation
PC: principal component
PCA: principal component analysis
PNN: probabilistic neural network
ROI: region of interest
TA: texture analysis.
